# Alientoma, a Dynamic Database for Alien Insects in Greece and Its Use by Citizen Scientists in Mapping Alien Species

**DOI:** 10.3390/insects12121101

**Published:** 2021-12-08

**Authors:** Konstantinos Kalaentzis, Christos Kazilas, Jakovos Demetriou, Evangelos Koutsoukos, Dimitrios N. Avtzis, Christos Georgiadis

**Affiliations:** 1 Sylvius Laboratory, Institute of Biology, Faculty of Science, Leiden University, 2333 Leiden, The Netherlands; konstakal95@gmail.com (K.K.); ckazilas@gmail.com (C.K.); 2 Naturalis Biodiversity Center, PO Box 9517, 2300 Leiden, The Netherlands; 3 Joint Services Health Unit Cyprus, BFC RAF Akrotiri, Akrotiri BFPO 57, Cyprus; jakovosdemetriou@gmail.com; 4 Section of Ecology and Systematics, Department of Biology, National and Kapodistrian University of Athens, 15772 Athens, Greece; vag18000@gmail.com; 5 Museum of Zoology, Department of Biology, National and Kapodistrian University of Athens, 15772 Athens, Greece; 6 Forest Research Institute, Hellenic Agricultural Organization Demeter, 57006 Vassilika, Greece; dimitrios.avtzis@fri.gr; 7 Section of Zoology and Marine Biology, Department of Biology, National and Kapodistrian University of Athens, 15784 Athens, Greece

**Keywords:** Alientoma, alien species, invasive species, citizen science, public engagement, Greece

## Abstract

**Simple Summary:**

Biological invasions have become one of the most intimidating environmental and economic threats of our time as a result of the globalisation and the rise in international commerce, with alien insects representing one of the most abundant groups of organisms introduced into Europe. Over the last decade, citizen science has emerged as a valuable tool for the early detection and monitoring of alien species worldwide. The aim of this study is to introduce a dynamic checklist and database of these organisms in Greece, where a large number of alien insect species have been detected. Alientoma—derived from “alien” and the Greek word “entoma”, meaning insects—was created to provide information on alien species (i.e., status, distribution, taxonomy, common names, and impacts) to the public as well as to the scientific community in order to inform and assist in the mitigation of their adverse impacts. This project was promoted through news agencies, both in the press and television, while it also maintained a strong social media presence. Since its launch, 1512 sessions were performed by individuals mainly from Greece and Cyprus. An initial network of citizen scientists has been established and is expected to grow in the near future.

**Abstract:**

Invasive alien species have been increasingly acknowledged as a major threat to native biodiversity and ecosystem services, while their adverse impacts expand to human health, society and the economy on a global scale. Insects represent one of the most numerous alien organismic groups, accounting for about one fifth of their total number. In Greece, a large number of alien insects have been identified, currently reaching 469 species. In recent decades, the contribution of citizen science towards detecting and mapping the distribution of alien insects has been steeply increasing. Addressing the need for up-to-date information on alien species as well as encouraging public participation in scientific research, the Alientoma website—derived from “alien” and the Greek word “entoma”, meaning insects, is presented. The website aims towards providing updated information on alien species of insects to the public as well as the scientific community, raising awareness about biological invasions and addressing their distribution and impacts inter alia. By maintaining a dynamic online database alongside a strong social media presence since its launch, Alientoma has attracted individuals mainly from Greece and Cyprus, interacting with the website through a total of 1512 sessions. Alientoma intends to establish a constantly increasing network of citizen scientists and to supplement early detection, monitoring and management efforts to mitigate the adverse impacts of alien insects in Greece.

## 1. Introduction

Biological invasions have led to a rapidly growing environmental crisis as a result of climate change and human-mediated activities such as globalisation and the constant rise in international commerce [1,2,3]. This excessive movement of people and goods has resulted in the introduction of alien species beyond their native range, affecting native biodiversity, ecosystem services, society, human health and economy on a global scale. Invasive alien species (IAS) have been deemed to be a major driver of biodiversity loss inter alia through interspecific resource competition with native species, predation, transmission of pathogens and ultimately inhibition of ecosystem services [4,5,6]. Regarding the socioeconomic implications, biological invasions pose a serious threat in agriculture and the urban landscape [7,8], fisheries and tourism [9,10], and public health [11,12,13].

The spread of invasive alien species has long been perceived as an impediment in biodiversity conservation, evidenced by a global need to tackle their spread and to mitigate their adverse impacts, which was expressed at the Convention on Biological Diversity (CBD) in 1992. In Europe, guidelines for “the prevention and management of the introduction and spread of IAS” were issued for EU State Members in EU Regulation 1143/2014. This influential document has led efforts in the prevention of spread, detection, prioritisation, management and eradication of IAS in the old continent [14,15].

The number of alien organisms in Europe currently reaches approximately 14,000 species, with insects accounting for about one fifth of their total number [16]. These species have managed to reach regions outside their natural range of distribution, either actively or passively, through their introduction as biological control agents, their escape from confinement or transport, and even as contaminants and stowaways [17,18,19,20]. Insects are well known for a series of direct and indirect impacts on the invaded habitats and indigenous organisms [21], human welfare [22], as well as society and the economy [23,24]. Even though the impacts of alien insects have been receiving more and more research interest (i.e., by identification of disastrous consequences for ecosystems and socioeconomic parameters [25,26,27]), the list of IAS of Union Concern includes just one insect: the Asian hornet *Vespa velutina nigrithorax* Buysson, 1905 [28,29,30].

The alien insect fauna of Greece was first assessed by Avtzis et al. (2017) [31], compiling a checklist for 266 species alongside analyses of feeding behaviour, invaded habitats and origin. This endeavour was supplemented by Demetriou et al. (2021), raising the number of alien insects in the country to 469. Further increases in the number of alien insects in Greece were predicted, with new records of alien species being constantly discovered. Conventional research methods have to be integrated with novel approaches such as interactively involving the public in biodiversity monitoring of alien species.

Public participation in scientific research has gradually emerged as an impactful source of information, providing data that ultimately supplement our knowledge on the presence, distribution, abundance, behaviour and impacts of alien species around the world [32,33,34,35]. Citizen science records have aided efforts in unravelling the distribution of injurious invaders such as *Chlorophorus annularis* (Fabricius, 1787), damaging bamboo furniture [36], the jasmine pest *Corythauma ayyari* (Drake, 1933) [37], the invasive oak processionary moth *Thaumetopoea processionea* (Linnaeus, 1758) [38] and the marmorated stinkbug *Halyomorpha halys* (Stål, 1855) [39]. Although the validity and taxonomical accuracy of observational data alongside spatiotemporal biases in biodiversity monitoring by citizen scientists have been regarded as drawbacks of such approaches [40,41,42,43,44], citizen science has been proved to assist biosecurity surveillances by detecting alien insects inter alia [45]. Recently, the role of citizen science in environmental policy-making decisions has been gaining increasing interest, highlighting the valuable public input in biodiversity monitoring of invasive species [46].

In Greece, citizen scientists and researchers have joined forces multiple times, reporting on the presence and distribution of alien insects. Nature enthusiasts have provided evidence for the first sightings of alien species in the country, such as the giant mantises *Hierodula tenuidentata* Saussure, 1869 [47]; *Sphodromantis viridis* Forsskål, 1775 [48]; the lantana plume moth *Lantanophaga pusillidactylus* (Walker, 1864) [49]; the feather-legged fly *Trichopoda pennipes* (Fabricius, 1781) [50]; and the broad-headed bug *Nemausus sordidatus* (Stål, 1858) [51]. Additionally, citizen science records have depicted the expansions in the range of and updated distributions of alien insects such as *Leptoglossus occidentalis* Heidemann, 1910, reaching Crete [52] seven years after its initial discovery on the Greek mainland [53] as well as the box-tree moth *Cydalima perspectalis* (Walker, 1859) [54], where public participation unveiled the infestation of native box-tree stands [8].

The growing necessity to accumulate information about alien insects around the globe and to mitigate their impacts has led to the emergence of a number of online databases over the years, such as DAISIE (Delivering Alien Invasive Species Inventories for Europe) [18] and EASIN (European Alien Species Information Network) [16]. These online tools have acted as an effective method to raise awareness and have greatly facilitated the monitoring of biological invasions [55,56,57]. The compilation of up-to-date information has acted as an important driver for the implementation of science-based policies in the past [58,59,60], yet these pre-existing projects are often not region-specific oriented. In acknowledgment of this need, a comprehensive information system for these organisms needs to be established on a national level.

In this publication, we introduce Alientoma—derived from “alien” and the greek word “entoma”, meaning insects—a project aiming to create a dynamic checklist and database of alien insects in Greece. Our goal is to promote public participation in scientific research of alien species by building an active community of citizen scientists able to identify and record alien insect species throughout Greece. Species occurrence records and relevant data submitted through the Alientoma data collection form will be used to monitor the spread of these species in Greece and to assist in the mitigation of their adverse impacts.

## 2. Materials and Methods

### 2.1. Construction of the Website

The catalogue of 469 alien insect species in Greece, which constitutes the backbone of this endeavour, was compiled after a thorough literature survey, review of museum collections and online databases [61]. In addition to the checklist, profiles of 50 species (an initial selection of easily recognisable species; all species were scheduled to be implemented) and details on their status, distribution, taxonomy, common names (also in Greek) and impacts were integrated into the website of Alientoma, which was created using the template for taxonomic websites of Scratchpads 2.0 [62]. Basic insect terminology, photographic material and specific diagnostic characteristics to distinguish them from their native or alien lookalikes were also provided for each species.

One of our approaches to promote the engagement of citizens in scientific research was adopting social media strategies. Social media have been recognised as an effective way for organisations to create interest and to build relationships with the public [63]. At the same time, maintaining a strong presence on the internet through our own social media pages allowed us to not only interact with citizen scientists on a higher level but also share educational material to a wider community and increase awareness. Shared material included taxon descriptions, identification keys, photographic material and notes on the ecology and the current distribution of the alien species. Moreover, public outreach efforts were made through news agencies, both in the press and television.

### 2.2. Web Analytics

Alientoma was registered with Google Analytics (GA) to monitor website traffic as well as insights on user preferences and serviceability. Although this service is primarily targeted for proprietary websites that provide a deeper understanding of customers visiting business websites, it is a free tool that can be also used for analysing visitors’ information for any website [64]. The major metrics utilised for Alientoma were users’ visits, origin and behaviour. Public interaction with the pages of the website was analysed by the Bounce rate, where “a bounce is calculated specifically as a session that triggers only a single request to the Analytics server, such as when a user opens a single page on your site and then exits without triggering any other requests to the Analytics server during that session” [65]. GA provides the opportunity to verify individual users based on the unique IP addresses and cookies acceptance registered in the system. Indeed, there is always the possibility that some of these users might be the same people logging in from different machines, but overall, this accounts for a small percentage of the true unique visitors of such websites [66]. Especially for behaviour, GA gives information regarding the sequence of pages visited per user during their visit. This metric offers an understanding on which pages are most accessed and thus indicates the path from which most users retrieve information.

### 2.3. Data Collection, Validation and Dissemination

Users can submit their observation data in a collection form provided in our website. After submission, photographic material, geographic coordinates, collection dates, contact details as well as the number of observed individuals are gathered and critically examined to ensure all necessary information are complete and the species of interest has been correctly identified. This form is easily accessible through our website and frequently provided by us under appropriate social media posts, so that citizens are aware of our database and can voluntarily share their records with us. Apart from the data collection form, citizen science records are also gathered from a separate project (https://www.inaturalist.org/projects/alien-insects-of-greece) hosted within the iNaturalist [67] umbrella project.

While citizen scientists’ participation is greatly encouraged, referred standards are to be strictly followed regarding data collection. Submitted data are validated by the authors using keys and species descriptions or comparing with museum specimens. For this, it is essential that the data (i.e., photographic material) are of high quality and depicting key characteristics of the species in question. In the case that species identification cannot be fully resolved using only photographic material, citizens are encouraged to provide a specimen for inspection, and only then is the record added in our database.

Collected data are archived, and their submission to GBIF is planned. Special attention is given to records of alien species new to Greece, and it is our effort that, upon meticulous examination, these are published in peer-reviewed scientific journals. Regarding records of species already included in the checklist, georeferenced maps depicting their up-to-date distribution will be shared through our website when enough data are collected. 

## 3. Results

### 3.1. Interface

**Figure 1:**
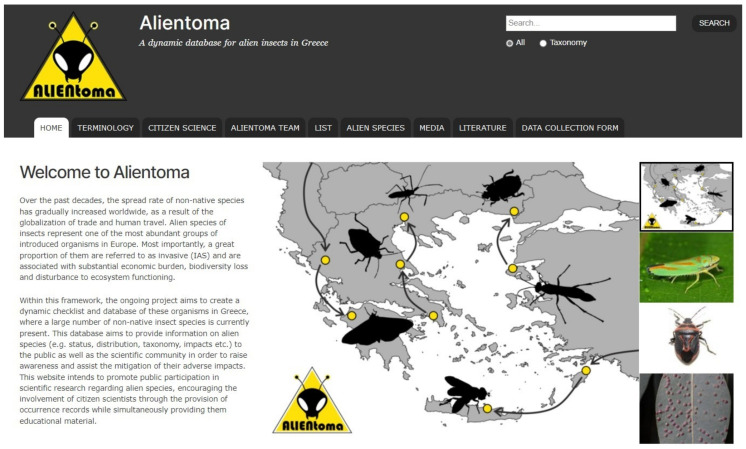
Main page of the Alientoma website.

The Alientoma website provides visitors with a wide variety of features. The home page provides a welcoming message highlighting the scope of the project (Figure 1). Citizen scientists and academics can easily navigate through eight additional website tabs. The public can familiarise themselves with the terminology used in invasion biology and learn about citizen science and its contribution to the scientific research of alien insects, through the corresponding tabs “Terminology” and “Citizen Science”. Visitors can learn more about the scientific community handling the database in the “Alientoma Team” tab.

The “List” section includes the current checklist of alien and cryptogenic insect species recorded in Greece, while “Alien species” provides a taxonomic overview of the alien insects of Greece; by clicking on each insect order, the families and subsequently species are shown. In each species profile, information is fed to four labels. In “Overview” (1), the nomenclature of the species, its common names, its first year recorded and published, the species status, photographic material and an occurrence map retrieved from GBIF are depicted. Under “Description” (2), the species morphology, identification from similar looking species, distribution, as well as ecology and impacts are discussed. The last two labels provide photographic material (3) and the literature (4) utilised during text preparation for each species. The latter two features can also be accessed through the home page under the “Media” and “Literature” tabs, respectively. Finally, citizen science records are submitted in the final tab under the respective name.

### 3.2. Web Analytics

Shortly after its launch (22 May 2021; International Day for Biological Diversity), the website was registered to the web analytics service offered by Google that tracks and reports website traffic Google Analytics (GA). Since then, GA has managed to pinpoint some interesting facts regarding the use and visibility of Alientoma to the general public. Until today (19 October 2021), 1036 individual users have visited the website, registering 1512 sessions and visiting 7246 pages (4.79 pages/session). Of those visitors, 14.1% have at some point returned to the website. Most of the new users visited the website almost immediately after its launch, with a peak of 101 individuals visiting the website on May 22, after the advertisement of the website on several social media pages.

**Figure 2:**
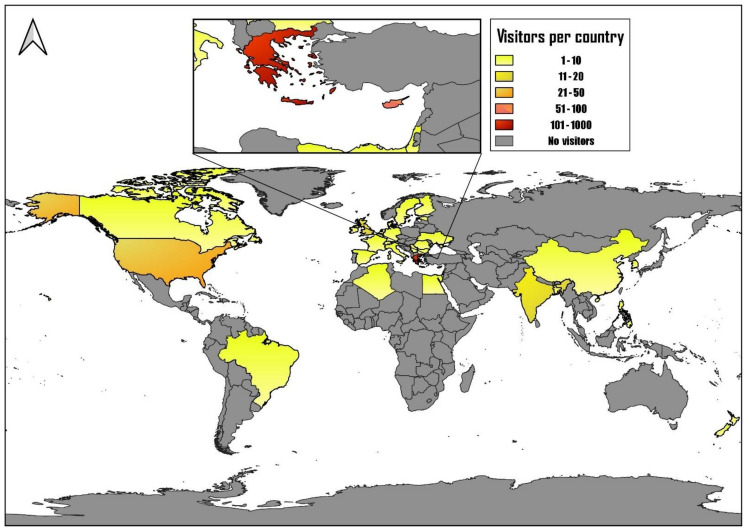
Alientoma users’ country map.

The vast majority of the users of Alientoma are from Greece (849 users), with users from Cyprus (65), the United States (23) and India (14) following (Figure 2). Analysing their behaviour, almost half of the users had no interaction with the website but 51.92% indicated some interaction with at least one of the website’s pages. The Bounce rate showed 0%, with sessions from Denmark, Belgium, Ukraine (1 session) and New Zealand (3 sessions). Regarding the users showing the highest number of visits, the bounce rate was 47.24% for Greece, 39.68% for Cyprus, 69.57% for the US and 64.29% for India, meaning that especially the latter ones were mostly random, accidental visits to the website. When looking into the users’ interaction flow, we see that the majority of the users holds interaction to at least 4 pages per session, with the maximum pages per session reaching 12.

### 3.3. Preliminary Results of the Data Collection Form

Until today, 54 records have been submitted through the Alientoma data collection form by 38 citizen scientists from all geographic regions of Greece with the exception of the Ionian Islands. Upon validation, 18 species of alien species were identified, while one record concerned a misidentified native species. Among the 18 observed species, the leafhopper assassin bug *Zelus renardii* Kolenati, 1856, was the most frequently observed, with 11 records, followed by the box-tree moth *Cydalima perspectalis* (Walker, 1859), with 6 records, and by the Asian ladybird *Harmonia axyridis* (Pallas, 1773) and the giant Asian mantis *Hierodula tenuidentata* Saussure, 1869, with 5 records each.

## 4. Discussion

In recent decades, citizen science has been rapidly growing largely due to technological advances (i.e., smartphones and GPS), providing vast sums of data regarding alien species [34]. These data constitute an important contribution towards understanding biological invasions and addressing the drawbacks of citizen science compared with traditional and novel surveillance methods [68]. The identification of species throughout photographic material has been regarded as a weakness of citizen science methods [40,41], although participants can be educated and trained in promptly and accurately identifying alien species [44,69]. In the case of Alientoma, species recognition relies on their robust identification through photographic material attached to the “Data collection form”. Thus, morphologically distinctive taxa such as *Xylotrechus chinensis* (Chevrolat, 1852) recently spreading from Crete to Continental Greece [70] or taxonomically unique species, i.e., *Hermetia illucens* (Linnaeus, 1758) [71], can be easily recorded and monitored. However, in the case of taxa with ambiguous taxonomy, experts are consulted to ensure high taxonomic accuracy. Furthermore, the uploaded species profiles provide a description of the species and how they can be distinguished from native counterparts or morphologically similar-looking species. This information can promote biodiversity monitoring training for citizen scientists, leading to the provision of less doubtful data and photographs where diagnostic morphological characters of alien species are evident [72].

Data mining and monitoring of alien species throughout social media has been also increasing in popularity [73,74]. The social media page constructed for Alientoma on Facebook promotes public participation in the study of alien insects of Greece by encouraging citizen scientists’ involvement, promoting educational material and assisting in the identification of uploaded material. This direct form of communication with citizen scientists contributes towards raising public awareness about alien species, forming an initial community of citizen scientists and shedding light on the impacts and public perception of observed alien insects [71,75].

The establishment of an efficient early warning system has been stated as a necessary step towards mitigating the future impacts of alien insects in Greece [61]. Such a scheme for insects has yet to be found in the country and will be integrated to the Alientoma website in the near future. Initiatives regarding marine invasions have been already implemented in Greece, providing multiple novel records for alien species detected by volunteers, shell collectors, fishermen and other citizens [76,77]. Alien insects with a high probability of arrival will be pooled in a separate section, indicating possible future invaders from nearby European countries and trading partners [78]. Alien insects of great economic significance and indisputable morphology such as *Anoplophora chinensis* (Forster, 1771); *A. glabripennis* (Motschulsky, 1854); or the small hive beetle *Aethina tumida* Murray, 1867, will be targeted, aiming to alert the public about their introduction risks. Educational material (species profiles, posters and identification guides) highlighting the morphology, ecology, distribution and impacts of these species will be constructed, raising public awareness. Data collection for possible propagules will be mediated through the data collection form and social media profile. This section aims to elicit feedback through the participation of citizen scientists, enhancing the possibility of early detection of an introduced species [69].

Regarding the web analytics, the large Bounce rate for Greece, of almost 50%, is rather troubling. Although it is understandable that not everyone is interested in biological invasions or insects, the website was mostly advertised in biodiversity-recording social media pages and media reporting on environmental issues. Thus, future Alientoma endeavours should focus on catching the eye of website visitors, addressing their interests and finding out what they are seeking when visiting the website (e.g., identification guides, information about species, health risks or socioeconomic impacts) and will provide interactive teaching activities through game-based learning, short videos, and individual acknowledgement of their contributions to enhance community building [46,79]. In addition, at a later stage, we will provide translation in English for the data provided per species to maximise visibility, accessibility and use for non-Greek speaking visitors.

Regardless of data collection, this database has set the creation of updated distributional maps for each species as a fundamental goal, where all collected data will be depicted. These maps will be open to the public for every means of scientific use, with the hopes of being an additional helping hand to citizen scientists, researchers and the public as whole, raising awareness and assisting future projects regarding the treatment of alien insects and their impacts.

## 5. Conclusions

The Alientoma database has managed to attract more than one thousand visitors in a 5-month period while hosting only a portion of the current checklist of 469 alien insect species. Public engagement is constantly increasing as a result of our promotion in both conventional and social media platforms. An initial network of citizen scientists, trained to identify and record alien insects throughout Greece, has been established and is expected to expand in the future when more species profiles and an early warning system are implemented in our database.

## Data Availability

http://alientoma.myspecies.info/el or/and https://alientoma.myspecies.info/en.
